# Gazelles, unicorns, and dragons battle cancer through the Nanotechnology Startup Challenge

**DOI:** 10.1186/s12645-016-0017-6

**Published:** 2016-06-09

**Authors:** Rosemarie Truman, Cody J. Locke

**Affiliations:** The Center for Advancing Innovation, INC., Bethesda, MD USA

## Abstract

On March 4th, 2016, Springer’s C*ancer Nanotechnology* office promoted the launch of the *Nanotechnology Startup Challenge in Cancer* (*NSC*^*2*^). This innovation-development model is a partnership among our company, the Center for Advancing Innovation (CAI), MedImmune, the global biologics arm of AstraZeneca, and multiple institutes at the National Institutes of Health (NIH). *NSC*^*2*^ “crowdsources” talent from around the world to launch startups with near-term, commercially viable cancer nanotechnology inventions, which were developed by the National Cancer Institute (NCI), the National Heart, Lung and Blood Institute (NHLBI), and the National Institute of Biomedical Imaging and Bioengineering (NIBIB). Crowdsourcing is a process in which one uses the internet to engage a large group of people in an activity, such as *NSC*^*2*^. For this initiative, CAI engaged universities, industry professionals, foundations, investors, relevant media outlets, seasoned entrepreneurs, and life sciences membership organizations to request that they participate in the challenge. From this outreach, fifty-six key thought leaders have enrolled in *NSC*^*2*^ as judges, mentors, and/or advisors to challenge teams (http://www.nscsquared.org/judges.html). Along with crowdsourcing talent to bolt startups around NIH inventions, *NSC*^*2*^ will also catalyze the launch of companies around “third-party” cancer nanotechnology inventions, which were conceived and developed outside of the NIH. Twenty-eight robust teams were accepted to the challenge on March 14th, 2016.

In total, 274 participants are competing in *NSC*^*2*^. These participants include life sciences industry players, serial entrepreneurs, angel investors, broadly trained scientists and engineers, experienced attorneys, and budding entrepreneurs with complementary backgrounds. Teams applied to the challenge through a Letter of Intent where they answered questions about their experience, role on the team and why they want to enter the challenge (Fig. 1). CAI evaluated these teams on more than forty criteria, which we developed through an analysis of successful startups. Each team has at least two currently enrolled students and one seasoned entrepreneur with more than 3 years in a startup. Twenty teams are based in the United States, three are based in China, two are based in Canada, two are based in England, and one is based in Brazil. Of the US teams, nine are based in the Maryland-Washington, DC-Virginia area.

**Fig. 1:**
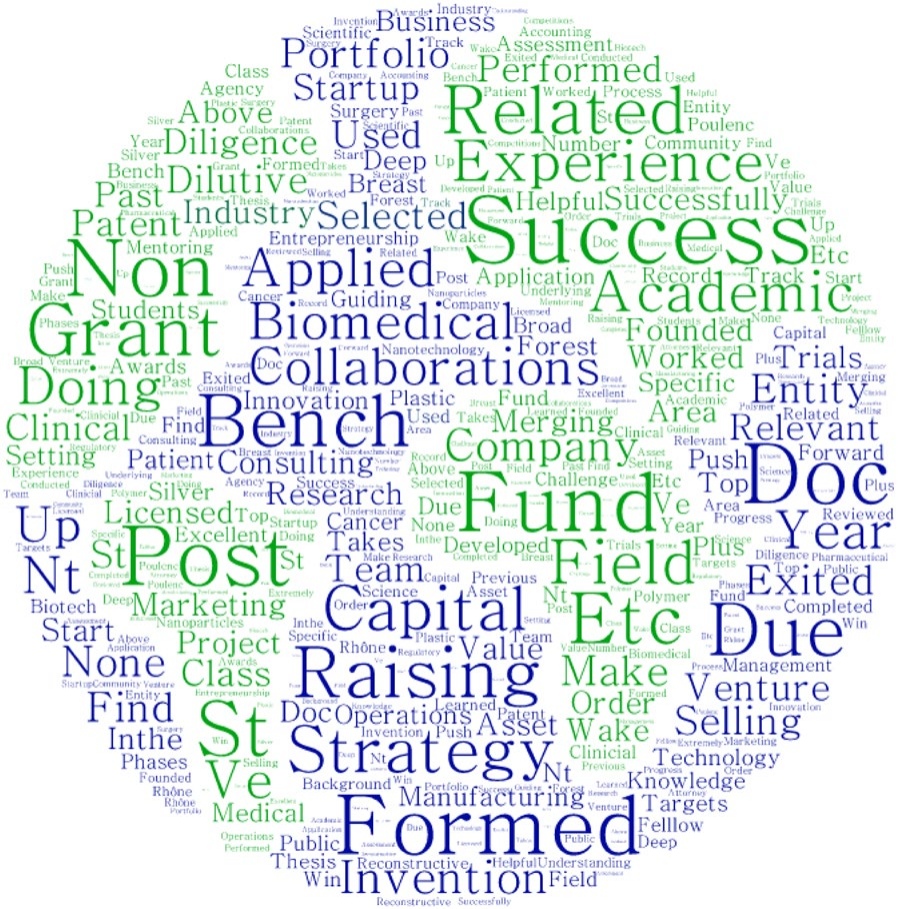
Word cloud map created by Rosemarie Truman based on NSC2 entrants answers to Letter of Intent questions upon entry into the challenge

Through *NSC*^*2*^, twenty-four teams are competing to license eight NIH inventions, whereas four teams submitted their own inventions with strong commercialization potential. Of the eight NIH inventions, three are drug candidates, three are diagnostics, and two have the potential of being therapeutics and/or diagnostics.

What outcomes do we hope to achieve through *NSC*^*2*^? This challenge, like our prior ones, is not simply a business plan competition. Our objective is to launch up to fifteen startups, which will commercialize the promising inventions in this challenge. The sheer impact that can be made through the *NSC*^*2*^ program is meaningful. First, nanotechnology itself is disruptive and could allow innovators to fine-tune existing cancer therapies without a need to develop new bioactive compounds. We hypothesize that nanotechnology will significantly decrease cancer-driven mortality rates, reduce the invasiveness of cancer diagnostics, and allow current therapeutics to target the cancer cells more precisely, thereby eliminating harmful side effects.

While making a timely and positive impact on human health, we also hope to create “gazelles” and potentially even “unicorns” or “dragons” from *NSC*^*2*^. Gazelles are companies that double revenue and jobs every 4 years. Unicorns are companies that earn a billion-dollar valuation within 5 years of being launched and 1.28 % of new startups have a chance of being a unicorn.  There are only 229 of unicorns; and, only 8 of them are life sciences related. Dragons, on the other hand, may not be valued at over $1 billion; yet, dragons are extremely profitable, returning all of the capital issued out of the fund—a “fund-maker.” In the startup world, these creatures are not mythical. However, they are rare. Dragons are four times rarer than unicorns, and only 27 % of unicorns are fund-makers. In addition to launching promising startups, we also will train hundreds of university students, post-docs, and others who have entered the challenge on the “business of science”. So far, CAI’s accelerator has trained 1000+ people through 40 classes, homework and hands-on experiential learning exercises that focus on how to develop business plans, create financial models, perform live pitches and raise money.

To create near-mythical startups, *NSC*^*2*^ teams will pursue a diversity of cancer nanotechnology inventions. The three exclusively therapeutic NIH inventions are photoactivatable liposomes for targeted drug delivery (Yavlovich et al. 2009, 2011; Puri et al. 2011), nucleic acid nanocubes for triggering RNA interference (Afonin et al. 2010, 2014a), and carbohydrate-encapsulated gold nanoparticles for inhibiting metastasis (Svarovsky and Barchi 2003; Brinãs et al. 2012). Seven, one and two teams will pursue these therapeutics, respectively. The three exclusively diagnostic NIH inventions are a carbon nanotube transistor-based microarray binding sensor (Subramanian et al. 2012), a DNA-tethered bead immunoassay (Silver et al. 2015), and an enzyme-catalyzed gold nanoparticle-based colorimetric assay (Liu et al. 2014). Six, one, and two teams will pursue these diagnostics, respectively. The remaining inventions are nucleic acid nanoparticles (Afonin et al. 2014b), which have been shown to induce RNA interference (Afonin et al. 2015), and polymer-coated gold nanorod assemblies for targeted, imaging-guided photothermal therapy (Rong et al. 2015; Song et al. 2015). Two and three teams will pursue these multifunctional inventions, respectively. One may find overviews of these eight inventions, offered through *NSC*^*2*^, at http://www.nscsquared.org/inventions.html.

We interviewed an *NSC*^*2*^ team leader, Elizabeth Cho-Fertikh, Ph.D. of Washington, DC, about her participation in this challenge. She noted, “I entered the challenge having been on the research and investor sides for many years, but never as an entrepreneur. The greatest value I have obtained so far from *NSC*^*2*^ is developing a venture with so much support in a structured and well-organized manner from the sponsors, obtaining pearls of wisdom throughout the process.” Dr. Cho-Fertikh’s team will attempt to commercialize a third-party peptide-based approach to target alternatively activated M2 macrophages (Cieslewicz et al. 2013; Ngambenjawong et al. 2016).

Phase 1 of *NSC*^*2*^ ended on April 17th, 2016. Each team submitted a two-minute “elevator speech”, which summarizes their chosen invention and potential startup, for public access and voting. Public voting was held between April 18th and April 22nd. The elevator speeches can be viewed at https://www.youtube.com/playlist?list=PLvu6n-GsGFsYNp-TFHeYOrB_bpaYYrjHM. Although public voting has closed, we would appreciate additional comments, likes, and shares of these elevator speeches. Feedback from the readers of *Cancer Nanotechnology* will be valuable to *NSC*^*2*^ participants and would serve to further the educational goals of this NIH-supported initiative. CAI announced finalists from Phase 1 on April 25th. After moving on to Phase 2, teams will write ten-page business plans, create robust financial models, and pitch to our panel of judges. The *NSC*^*2*^ teams may continue to add teammates through the challenge. To join an existing team, visit http://www.nscsquared.org/find-a-team–member.html.

You can find out more about each of the inventions from the references list below. We are most grateful to the *Cancer Nanotechnology* editorial team for helping to promote *NSC*^*2*^. Stay tuned for a Special Collection of articles, related to the inventions in *NSC*^*2*^ and authored by the teams.
